# The high cost of healing and teaching: a cross-sectional survey of burnout among academic physicians in Nigeria

**DOI:** 10.1186/s12913-023-10366-1

**Published:** 2023-12-05

**Authors:** Kehinde Sunday Oluwadiya, Anthony A. Olasinde, Adekunle Olatayo Adeoti, Oyewole Adeoye, Ibironke Omowunmi Oluwadiya, Innih Asuekome Kadiri

**Affiliations:** 1 Department of Surgery, University of Sierra Leone, Freetown, Sierra Leone; 2 Department of Surgery, Kampala International University (Western Campus), Ishaka-Bushenyi, Uganda; 3 Department of Medicine, Ekiti State University, Ado-Ekiti, Ekiti State Nigeria; 4 Department of Psychiatry, Ladoke Akintola University of Technology, Ogbomosho, Oyo State Nigeria; 5 Department of Demography and Social Statistics, Obafemi Awolowo University, Ile Ife, Osun State Nigeria; 6 Department of Surgery, Ekiti State University, Ado-Ekiti, Ekiti State Nigeria

**Keywords:** Burnout, Academic physicians, Prevalence, Nigeria

## Abstract

**Background:**

Globally, the medical and teaching professions are two major professions with the highest prevalence of burnout, and academic physicians bestride the two professions. This study investigated the prevalence and associated factors of burnout among academic physicians working in tertiary hospitals in Nigeria.

**Methodology:**

This was a self-administered online survey. Burnout was measured using the Maslach Burnout Inventory for Educators (MBI-ES) on Google Form and sent to 256 academic physicians in tertiary hospitals across Nigeria using the WhatsApp broadcast feature. MBI-ES was categorized into two categories (Burnout and No Burnout), and binary logistic regression was used to test the influence of 13 predictors on the three dimensions of MBI-ES as well as MBI in its entirety.

**Findings:**

A total of 155 academic physicians responded, resulting in a response rate of 60.5%. There were 121 (80.7%) males and 29 (19.3%) females (five cases respondents omitted this detail). Eighty-seven respondents exhibited moderate to high burnout in at least one of the dimensions of the MBI, translating to a prevalence rate of 57.7% in our study. Five variables, number of peer reviewed articles published, hours of weekly teaching, enjoyment of academic writing, apathy to teaching and religion were all significantly associated with burnout. Moderate to high emotional exhaustion was reported by 30.8% (45 respondents), moderate to high depersonalization by 5.5% (8 respondents),, and low to moderate personal accomplishment by 43.5% (67 respondents).Eight variables: religion, geopolitical zone of practice, enjoyment of academic writing, apathy toward teaching, university ownership, number of published peer-reviewed articles, salary, and supplementary income were significantly associated with emotional exhaustion, while the number of weeks spent teaching in a year and teaching hours/week were significantly associated with depersonalization and personal accomplishment, respectively. Age (OR 1.302, CI 1.080–1.570), Teaching hours/week (OR 0.924, CI 0.854–0.999), Salary (OR 0.996, CI 0.993-1.0), and supplementary salary (OR 0.996, CI 0.993–0.999) were found to significantly predict emotional exhaustion.

**Conclusion:**

The study reveals a high prevalence of burnout (57.7%) among academic physicians in Nigeria, highlighting an urgent need for targeted interventions and policy changes. Given the significant role these professionals play in healthcare and medical education, immediate action is essential to address this issue. Future research should focus on evaluating the effectiveness of preventive measures and exploring the long-term impacts of burnout.

## Introduction

Burnout is typically characterized by high levels of emotional exhaustion, high levels of depersonalization, and low levels of personal accomplishment resulting from prolonged exposure to job stressors [1]. Emotional exhaustion refers to the feeling of being overwhelmed and emotionally drained by one’s work [1, 2]. Depersonalization is the adoption of a callous or dehumanized perspective towards others, and it is often a result of emotional exhaustion [1–3]. Low personal accomplishment is the feeling of dissatisfaction with one’s professional achievements [1–3].

Physician burnout has gained increasing recognition as a major problem in the healthcare industry worldwide [2, 4–6]. Many factors contribute to this phenomenon, including reduced financial compensation, longer work hours, medicolegal issues, and a decline in the status of physicians within the healthcare system [2, 6–8]. Additionally, academic physicians have the added responsibility for teaching, grading, and mentoring students, which may make them more susceptible to this problem [2, 3]. In addition to cultural factors, Lee et al. identified two important categories of environmental factors that can influence physician burnout: environmental drivers and environmental constraints. Environmental drivers promote work engagement and well-being, whereas environmental constraints can lead to emotional exhaustion or burnout. In their systematic review, they found that American physicians experience lower levels of emotional exhaustion than their European counterparts, primarily due to the fact that their work environments offer more environmental drivers and fewer environmental constraints [9]. It is important to distinguish burnout from job apathy and depression. Schmidt et al. have identified two types of apathy: clinical and selective [10]. Clinical apathy is defined as a lack of motivation which affects all areas of life, while selective apathy is characterized by a lack of interest, activity, and emotional involvement in a specific domain of life [10]. Job apathy is a type of selective apathy that is related to one’s job. In this vein, some authors have identified job apathy as one of the burnout stages [11, 12]. Both authors identified job apathy as the final stage of burnout. Depression, on the other hand, is nearly identical to burnout, with the exception that burnout is workplace-related, whereas depression is widespread.

There are numerous adverse effects of burnout. In addition to the emotional distress associated with the condition, it can result in decreased productivity, job dissatisfaction, illnesses, and substandard patient care. According to studies, burnout can also be contagious as it can spread through the ranks of employees in hospitals and academic departments [3].

Compared to other parts of the world, there have been very few reported studies on burnout among physicians from Africa in general and Nigeria in particular [7, 13]. Reported prevalent range of the disease in Nigeria ranges between 23.6% and 75.5% [6, 14]. In comparison to their counterparts in High-Income Countries (HIC), Nigerian physicians may be more vulnerable to burnout due to low income and poor job satisfaction caused by a poor work environment and a lack of facilities for them to practice their skills [15]. Therefore, the added frustration of powerlessness when faced with patient suffering, despite having the skill to treat the patient, if the necessary facilities are available, is most likely a powerful enabler of burnout among Nigerian physicians. Burnout has also been identified as a significant contributor to the physician brain drain currently afflicting the Nigerian health sector [16].

This study examined the characteristics of burnout among academic physicians in Nigeria. Additionally, we investigated the impact of sociodemographic and professional variables, in addition to stressors, on burnout among the cohort.

## Patients and method

### Study design and participants

An online questionnaire-based survey of academic physicians was carried out among academic physicians in Nigeria between December 2022 and January 2023 using Google Form as the tool and WhatsApp as the distribution platform. Nigeria has 36 states and a federal capital territory divided into six geopolitical zones: Southwest, Southeast, Southsouth, Northwest, Northeast and North Central. For the purposes of this study, an academic physician is a consultant doctor who is a fellow of either or both postgraduate medical colleges in Nigeria or their equivalent and whose main job is to teach at a medical school while also working as a consultant in a teaching hospital. Frequently, the individual will also be responsible for teaching resident doctors. Therefore, an academic physician combines clinical and academic responsibilities. They may be part-time or full term, meaning they are fully employed by the university. Their ranks range from Lecturer 1 to Senior Lecturer, Associate Professor, and finally Professor.

### Survey administration

We created a Google form and distributed its link to academic physicians via the WhatsApp’s broadcast feature. Using the WhatsApp broadcast feature, we sent the Google form to 256 recipients. This method was chosen because it permitted us to calculate the response rate. In addition, the method guaranteed anonymity and permitted us to provide study participants with feedback. Participants were randomly selected from the WhatsApp group of the Medical and Dental Consultants’ Association of Nigeria (MDCAN), the umbrella organization for all specialists in the country. In Nigeria, while only consultants are eligible to serve as clinical lecturers in medical schools, not all consultants hold lecturing positions, so the MDCAN WhatsApp platform includes both academic and non-academic specialists. The inclusion criterion for this study was that the consultant must also be a university lecturer. Initially, we sent a message to all members of the group, asking whether they were university lecturers and if they would be willing to participate in the study. Following repeated reminders, we successfully recruited the required 256 participants to form the broadcast group. Confidentiality was maintained by not including any identifying information in the questionnaires. Participation in the study was completely voluntary. The protocol and survey instrument were reviewed and approved by the Ekiti State University Teaching Hospital Institutional Review Board.

### Survey instrument

The survey instrument consisted of 46 questions divided into two main sections as follows:

Section One: Professional and socio-demographic information. Five questions were asked to collect basic demographic information, including age, sex, marital status, religion, and geopolitical zone. Four additional questions collected information on professional practice, including the year of graduation, the year the individual began academic teaching, their medical school, and whether the individual is employed by a private or government institution. Five questions addressed potential stressors, including income, research output, side jobs, other administrative duties, and academic load, which was measured by asking respondents how many hours per week they spent instructing medical students. Finally, their attitude toward academics was evaluated by asking them three questions: “Would you consider yourself a person who enjoys teaching?, “Would you consider yourself a person who enjoys writing academic papers?“ and “Would you consider yourself indifferent to clinical teaching/lecturing?“

Section Two: Maslach Burnout Inventory (MBI) was used to study burnout among university educators in Canada, [3], and has been validated in Nigeria [17, 18]. This questionnaire contains 22 questions that address the three components of burnout: nine questions on emotional exhaustion (EE), five questions on depersonalization (DP), and eight questions on personal accomplishment (PA). Respondents were then asked to rate the frequency of encountering these situations on a 7-point Likert scale ranging from “never” (0) to “every day (7).“ To determine a patient’s overall score for each subscale, we added the values of each item in the subscale that he or she ticked. For each subscale, the respondent is categorized into Low, Moderate, or High Burnout, as shown in Table 1. To obtain a high total score on burnout, you had to obtain high scores on emotional exhaustion (≥ 27), depersonalization (≥ 10),or a low score on personal accomplishment (≤ 33) [1, 2, 17]. At the end of this section, there was a place for general comments.

**Table 1:** Categorization of burnout subscales

	Emotional exhaustion	Depersonalization	Personal accomplishment
Low	≤ 18	≤ 5	≤ 33 ≥ 40
Moderate	19–26	6–12	34–39
High	≥ 27	≥ 13	≤ 33

### Statistical analysis

We performed a statistical analysis on data collected through a Google form. The data was downloaded in Microsoft Excel format and imported into IBM-SPSS Version 25. Discrete variables were compared using chi-square tests, while continuous variables were compared using t-tests.

A binary logistic regression was conducted to investigate the effect of 13 predictors on the overall incidence of burnout and subsequently on emotional exhaustion, depersonalization, and personal accomplishment dimensions. For this analysis, respondents who fell into the moderate to high categories according to Table 1 were classified as experiencing burnout, while those in the low category were deemed not to have burnout. The predictors were age, teaching experience, geopolitical zone of practice, university ownership (private, federal government-owned, state government-owned), number of published peer-review articles, sex, annual student contact in weeks, teaching hours/week, enjoyment of academic writing, apathy toward teaching, religion, monthly salary, and monthly supplementary income. These variables were selected based on findings from literature review and significant associations with the burnout dimensions in univariate analysis. The model selection was performed using a backward conditional method where all predictors were initially included and were iteratively removed based on a p-value threshold of 0.1. For the overall case, 10 models were tested with all of them demonstrating good fit (Hosmer Lemeshow Goodness of Fit test P > 0.05), but the Cox and Snell R-Square (0.297–0.408) and Nagelkerke R-Square (0.234 − 0.231) were low for all 10 models. Interestingly, models 1–3, which had the highest R-Square values failed to produce any statistically significant predictors, whereas models 7–10, each of which had one variable reaching significance, had much lower R-squared values than models 1–3. Given this situation, we decided not to select any of the models for the overall case of burnout incidence.

Regarding the logistic regression testing of the individual burnout dimensions, the models for depersonalization and personal accomplishment were rejected due to poor fit, as indicated by the Hosmer-Lemeshow Goodness of Fit test (P < 0.05). Only emotional exhaustion was further analyzed, as the Hosmer-Lemeshow test indicated that all eight models had a good fit (p > 0.05). A total of 20 iterations were performed, resulting in the removal of nine predictors and a final model with four models. Model 1 was selected as the best fit because it had the highest Cox and Snell R-square (0.374) and Nagelkerke R-square (0.571) values and it explained 82.1% of the variance in the model, which was one of the highest.

### Dataset

This is reposited on the Open Science Framework (OSF) Website [19].

## Result

Of the 256 participants invited to complete the survey through WhatsApp broadcast, 155 completed responses were received after weekly reminders over a 5-week period, yielding an overall response rate of 60.5%. The socio-demographic characteristics are shown in Table 2. The mean age of the respondents was 51.1 years (SD: 7.4 years), and the median years of teaching medical students was 12 years (range: 1–34 years). The participants spend a median of 12 h (range: 1–72 h) per week with the students and engage in teaching medical students for a median of 30 weeks (range: 2–54 weeks) per year.

**Table 2:** Socio-demographic characteristics of the respondents

Characteristics	No (%)
**Gender (n = 150)**	
Male	121 (80.7%)
Female	29 (19.3%)
**Academic Rank (n = 150)**	
Adjunct Lecturer	12 (8.0%)
Lecturer 1	24 (16.0%)
Senior Lecturer	42 (28.0%)
Associate Professor	30 (20.0%)
Professor	42 (28.0%)
**Private/Public University (n = 149)**	
Public	136 (91.3%)
Private	13 (8.7%)
**Geopolitical Zone(n = 149)**	
North	48 (32.2%)
Southeast	19 (12.8%)
Southwest	82 (55.0%)
Specialty (n = 148)	
Surgical	72 (48.6%)
Nonsurgical	55 (37.2%)
Dentistry	21 (14.2%)
**Other Sources of income (n = 126)**	
Yes	97 (66.4%)
No	49 (33.6%)
**Holds Administrative Post (n = 150)**	
Yes	77 (51.3%)
No	73 (48.7%)

### Reliability

Cronbach’s alpha was 0.95 for the subscale measuring emotional exhaustion, 0.72 for the subscale measuring depersonalization, and 0.93 for the subscale measuring personal achievement, indicating high reliability. Personal achievement did not significantly correlate with either depersonalization (r = -0.115, p = 0.189, N = 132) or emotional exhaustion (r = -0.099, p = 0.260, N = 132). However, a moderate and statistically significant correlation was observed between depersonalization and emotional exhaustion (r = 0.460, p < 0.01, N = 143).

### Prevalence of burnout

**Fig. 1:**
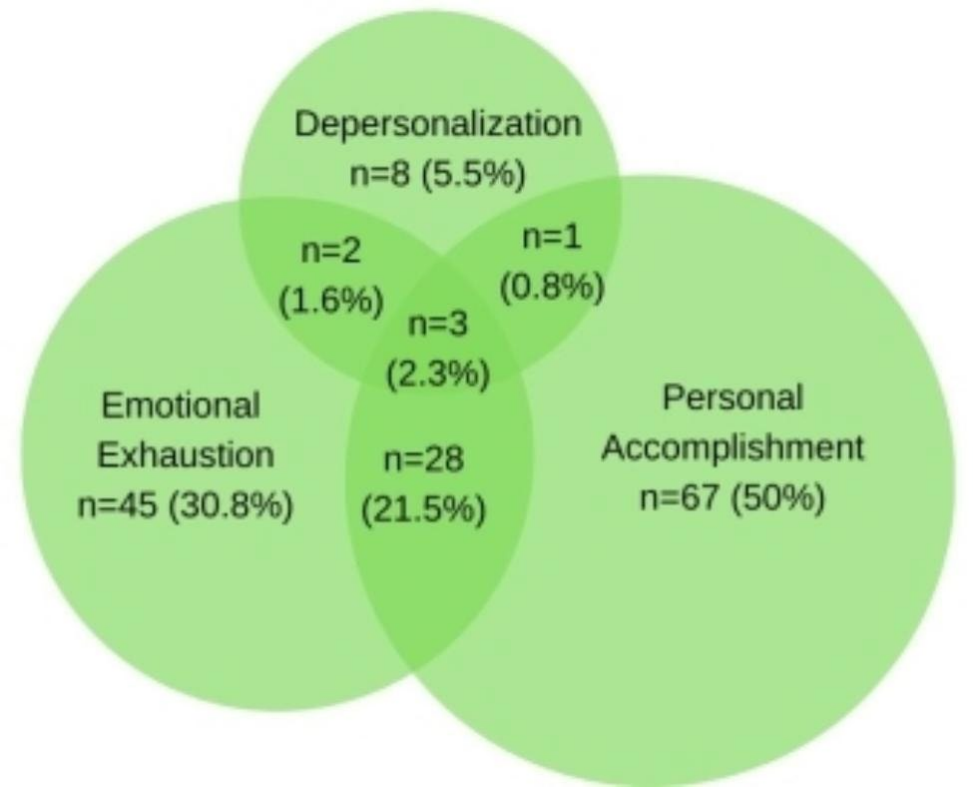
Proportion of respondents who reported moderate/high levels of depersonalization, emotional exhaustion, and moderate/low personal achievement dimensions of burnout among 130 respondents

The study found that 57.7% (86 participants) experienced moderate to high burnout in at least one of the three dimensions. Moderate emotional exhaustion was reported by 12.3% (18 respondents), high emotional exhaustion by 18.5% (27 respondents), moderate depersonalization by 4.8% (7 respondents), high depersonalization by 0.7% (1 respondent), moderate personal accomplishment by 10.4% (16 respondents), and low personal accomplishment by 33.1% (51 respondents). Figure 1 shows the proportion of academic physicians with moderate/high burnout and the overlap of burnout in the three dimensions. A total of 50% reported moderate-to-low personal accomplishment, 30.8% reported moderate-to-high emotional exhaustion, and 5.5% reported moderate-to-high depersonalization. Three respondents reported high levels of burnout in all three dimensions simultaneously, and the remaining 23.9% experienced burnout in two of the three dimensions, with the majority reporting a combination of low-to-high emotional exhaustion and low-to-moderate personal accomplishment.

### Association between burnout dimensions and demographic variables

The prevalence of the three dimensions of burnout was found to vary significantly across demographic variables, as shown in Tables 3 and 4. Regarding the overall incidence of burnout, three variables - enjoyment of academic writing, apathy to teaching and religion - were all significantly associated with burnout as a whole (P < 0.05). Those three categorical variables as well as geopolitical zone of practice and university ownership were significantly associated with emotional exhaustion (Table 3). For example, Muslims had a higher prevalence of emotional exhaustion than Christians, doctors in the North had the highest prevalence among the geopolitical zones, and those working in federal government-owned universities had the highest prevalence of emotional exhaustion compared with those working in private or state-owned universities. Gender did not show a significant association in the analysis, but when the data were analyzed with high and low burnout separated, a significant association was found between depersonalization and sex, with the two cases of high depersonalization being females. However, both depersonalization and personal accomplishment were not significantly associated with any variables. In the case of depersonalization, this is probably due to the small sample size (n = 8) for this subscale, which limits the power to detect associations.

**Table 3:** Association between categorical sociodemographic variables and burnout

	Emotional exhaustion		Depersonalization		Personal accomplishment		Overall		
	n (%)	p-Value	n (%)	p-Value	n (%)	p-Value	n (%)	p-Value	
**Gender**									
Male	32 (27.4%)	0.068	5 (4.3%)	0.203	52 (49.1%)	0.547	65 (64.6%)	0.082	
Female	13 (44.8%)		3 (10.3%)				21 (72.4%)		
					15 (55.6%)				
**Marital Status**									
Married	42 (30.0%)	0.583	8 (5.8%)	0.833	65 (50.8%)	0.599	82 (57.7%)	0.49	
Single	2 (50.0%)		0				3 (75.0%)		
					2 (50.0%)				
**Religion**									
Christianity	28 (24.8%)	**0.003**	5 (4.4%)	0.279	51 (49.5%)	0.713	61 (53.0%)	**0.02**	
Muslim	17 (51.2%)		3 (9.4%)		16 (53.3%)		25 (75.8%)		
**Zone**									
Southwest	17 (21.3%)	**0.013**	3 (3.7%)		32 (44.4%)	0.256	41 (50.6%)	0.055	
North	21 (45.7%)		3 (6.5%)		27 (60.0%)		11 (57.9%)		
Southeast	7 (38.9%)		2 (10.5%)		8 (53.3%)		34 (72.3%)		
**Enjoy Writing**									
Yes	26 (25.7%)	**0.046**	5 (4.9%)	0.517	45 (47.9%)	0.37	53 (51.5%)	**0.013**	
No/Maybe	19 (42.2%)		3 (7.0%)		22 (56.4%)		33 (73.3%)		
Apathy to Teaching									
Yes	12 (66.7%)	**0.001**	1 (5.6%)	0.998	11 (64.7%)	0.153	17 (94.4%)	**0.001**	
No/Maybe	33 (24.4%)		7 (5.5%)		47 (47.8%)		67 (52.8%)		
**Academic Rank**									
Professor	18 (25.4%)	0.164	4 (5.6%)	0.952	29 (45.3%)	0.261	36 (50%)	0.052	
Non-Professor	27 (36.0%)		4 5.4%)		39 (55.1%)		50 (65.8%)		
**University Ownership**									
Federal	32 (42.1%)	**0.006**	7 (9.2%)	0.115	38 (49.3%)	0.983	50 (64.9%)	0.174	
State-owned	12 (21.1%)		1 (1.7%)		25 (51.0%)		29 (49.2%)		
Private	1 (7.7%)		0		6 (50.0%)		7 (53.8%)		
**Specialty**									
Surgical	23 (33.8%)	0.72	3 (4.3%)	0.655	34 (55.7)	0.247	42 (50.0%)	0.323	
Non-Surgical	15 (27.3%)		3 (5.7%)		22 (41.5%)		29 (52.7%)		
Dental	7 (33.3%)		2 (9.5%)		11 (57.9%)		15 (71.4%		
**Admin Position**									
Yes	21 (27.3%)	0.327	4 (5.2%)	0.856	36 (48.6%)	0.655	44 (57.1%)	0.804	
No	24 (34.8%)		4 (5.9%)		31 (52.5%)		42 (59.2%)		

Three continuous variables, the number of peer-reviewed articles published, salary, and supplementary income, were significantly associated with emotional exhaustion (Table 4). Meanwhile, the number of weeks spent teaching in a year and teaching hours/week were significantly associated with depersonalization and personal accomplishment, respectively. Also, only the number of published peer-reviewed papers and weekly hours of teaching were significantly associated with the overall level of burnout.

**Table 4:** Association between continuous sociodemographic variables and burnout

	Emotional Exhaustion		Depersonalization		*Personal Accomplishment		Overall	
	Mean	p-Value	Mean	p-Value	Mean	p-Value	Mean	p-Value
**Age**								
Low	51.4yrs	0.789	51.2yrs	0.387	50.8yrs	0.201	52.2yrs	0.136
Mod/High	50.3yrs		49.38yrs		51.0yrs		50.4yrs	
**Teaching experience**								
Low	13.1yrs	0.495	13.1yrs	0.965	12.2yrs	0.326	14.3yrs12.2yrs	0.086
Mod/High	12.5yrs		12.9yrs		14.0yrs			
**Papers Published**								
Low	42	**0.03**	39	0.464	34	0.887	45	**0.008**
Mod/High	31		36		43		33	
**Salary (000)**								
Low	554.6	**0.003**	510.9	0.636	476.1	0.839	565.8	0.064
Mod/High	433.1		553.9		533.6		472.3	
**Complementary Salary (000)**								
Low	529.6	**0.014**	452.3	0.295	431.8	0.475	519.8	0.383
Mod/High	346.6		694		468.1		434.3	
**Weeks/year teaching**								
Low	27.1	0.062	28.1	**0.008**	31	0.111	28.6	0.982
Mod/High	31.8		40.8		27.1		28.5	
Teaching hours/week								
Low	17.3	0.373	16.2	0.521	19.2	**0.02**	20.3	**0.018**
Mod/High	15.6		23		13.3		14.7	

*Please note that Low Level of Personal accomplishment connotes burnout and vice versa

### Risk factors for burnout

Out of the 13 variables considered in the binary logistic regression analysis, only four were found to have a statistically significant effect on the outcome. These variables were: Age, weeks spent teaching, salary, and supplementary income (p < 0.05). The results showed that a 1-year increase in Age increased the odds of high emotional exhaustion by 1.302, while a 1-week increase in the number of weeks spent teaching decreased the odds by 0.924. Furthermore, each 1000 Naira increase in salary and supplementary income reduced the odds by 0.004 (Table 5).

**Table 5:** Multivariate logistic regression for risk factors for emotional exhaustion

Variable	Adjusted Odds Ratio	95%CI	p-Value
Age	1.302	1.080–1.570	0.006
Teaching hours/week	0.924	0.854–0.999	0.048
Salary	0.996	0.993-1.0	0.046
Supplementary salary	0.996	0.993–0.999	0.014

## Discussion

This study investigated the prevalence and associated factors of burnout among academic physicians working in tertiary hospitals in Nigeria. Despite previous research on burnout in different groups of healthcare professionals, including all physicians, [4] resident doctors, [17, 20] specialists, [21] all hospital staff, [22] and medical students, [23] this is the first study to focus specifically on academic physicians in teaching hospitals and other tertiary healthcare facilities in Nigeria. This population is crucial to examine as they confront unique stressors from both patient care and medical student education.

One significant challenge in comparing the prevalence of burnout as reported by different researchers is the lack of a universally accepted definition for burnout. Some authors define burnout as concurrently reporting high levels of burnout across all three subscales: exhaustion, depersonalization, and reduced personal accomplishment [14]. Studies using this definition often report typically low burnout prevalence. For instance, Ozumba et al. reported a prevalence rate of 4.7% among Nigerian doctors and nurses [14]. Similarly, Al Dubai et al. reported a prevalence rate of 11.7% from Yemen [24]. In our research, consistent with the majority of authors, we characterized burnout as the manifestation of moderate to high burnout in at least one of the burnout dimensions [6–8, 14, 17, 25]. The results of this study indicate a high incidence of burnout among academic physicians, with 57.7% experiencing one form of burnout or another. Although this falls within the range of what has been reported from Nigeria [6, 14], Africa [7], and globally [8, 25], it is still concerning as burnout at any level is concerning.

### Emotional exhaustion

Of the three dimensions of burnout, emotional exhaustion is widely recognized as the best single measure for physician burnout [2, 3]. This study supports this finding as it is the only one with more than one significantly associated variable, and its model was significant after binary logistic regression. Many studies have explored the impact of religion on burnout among healthcare workers and have found that religion generally has a protective effect on burnout [26, 27]. However, few studies have compared the effects of different religious affiliations on burnout. Haghnegahdar et al. found no association between religious affiliation (e.g., Christian, Muslim, Atheist, Buddhist) and burnout among US medical students [28]. However, the same study showed that self-perception as an active participant in one’s religion was associated with lower burnout scores. Our study found a strong association between religion and emotional exhaustion, with Muslims more likely to report moderate to high emotional burnout than Christians. We cannot provide an explanation for this, but a systematic review of burnout across Arab countries by Elbarazi et al. reported a prevalence rate of 13.3–85.8% [8]. Only a few studies from multireligious or Christian-dominated countries have reported such high prevalence rates.

A surprising finding was that physicians working in Federal government-owned hospitals were more likely to report emotional exhaustion. In Nigeria, it is commonly believed that working for the Federal government is better than working for state governments, and employees are often poached from state government institutions to federal government institutions [6]. This is supported by Lar Ndam’s study of family physicians in Jos, Nigeria, which found a higher burnout prevalence among those working in state hospitals compared to those working in Federal government-owned teaching hospitals [29]. Perhaps this surprising finding is related to the fact that the study was conducted during a strike of lecturers working in federal government-owned universities, during which the Federal Government had not paid the striking lecturers for several months.

We also found that those practicing in the north were more likely to report moderate to high emotional exhaustion. This may be because a higher proportion of those practicing in the north are Muslims and work in Federal government-owned institutions. However, we were unable to explore this relationship further as our study lacked the power for such subgroup analysis. Future studies in Nigeria should consider examining this.

Despite the unique stressors faced by academic physicians, such as administrative tasks, increased academic workload, and balancing clinical, teaching, and research responsibilities, previous studies have shown that they are less likely to experience burnout compared to their non-academic counterparts (Norvel, Zhuang) [30, 31]. However, these studies failed to consider those who do not enjoy certain aspects of academic responsibilities. Apathy towards specific job duties can contribute to burnout, whereas engagement in enjoyable activities may provide a protective effect [30, 32]. Tijdink et al. found an association between publication pressure and a higher incidence of burnout among Dutch medical professors, and Norvel found that engaging in what one considers meaningful work was protective [32]. Our study confirms these findings, as apathy toward teaching and enjoyment of academic writing were both associated with burnout, indicating the added stress faced by academic physicians compared to their nonacademic colleagues. Further studies should explore this further.

The results of the logistic regression analysis produced two unexpected findings in this study. First, age was found to be a positive predictor of burnout which is in contrast to almost all the studies reported from Nigeria which showed burnout to be more common among the younger age groups [4, 6, 14, 17, 29]. This difference in results may be due to the fact that our study focused on physicians who have completed their specialist training, excluding those in the early stages of their careers. For example, the youngest participant in this study was 36 years old, which eliminates the influence of younger respondents.

The second unexpected result from the logistic regression analysis was that teaching hours per week had a negative impact on burnout, contrary to the assumption that increased workload would result in more stress. However, academic physicians may view teaching as both meaningful and crucial to their work. One study found that physicians who dedicate at least 20% of their time to activities they find most meaningful have a lower risk of burnout [30, 33]. Additionally, Golub et al. posited that many academic physicians choose to work in academic medicine because they love it and thus have different desires and motivations apart from their clinical activities [2] Further research is necessary to delve into these surprising findings.

### Depersonalization

The prevalence of depersonalization in this study was low compared to other studies conducted in Nigeria, which reported a prevalence ranging from 21 to 57% as reported by Ogunsiji et al. in their systematic review of burnout in Nigeria [6]. This difference may be due to the study population being post-fellowship consultant physicians who belong to the highest cadre in the medical profession and have a high degree of control over their work environment. Control over work tasks and decision-making has been linked with lower levels of depersonalization [34].

### Personal accomplishment

The dimension of personal accomplishment recorded the highest level of burnout in this study, which is consistent with the findings of the study by Ogundipe et al. [17]. This could be attributed to the poor economic climate in the country, as well as the national strike by lecturers at the time of the study, as a result of which the salaries of lecturers working in federal government-owned hospitals were not paid for many months. Poor salary can result in feelings of frustration and a sense of being undervalued, which can negatively impact one’s sense of personal accomplishment and influence their motivation and satisfaction with work [6].

## Conclusion

The study investigated the incidence of burnout among academic physicians in Nigeria and found that nearly three out of five experienced burnouts, with personal achievement being the most commonly reported dimension. The study also found that religion and location of practice had an impact on emotional exhaustion, with Muslims and those practicing in the North more likely to report moderate to high levels of burnout. Additionally, apathy towards teaching and enjoyment of academic writing were also associated with burnout. The results of logistic regression analysis showed unexpected findings, with age being a positive predictor of burnout and teaching hours per week having a negative effect. The study highlights the need for further exploration into the unexpected findings and the added stress faced by academic physicians in Nigeria.

Understanding the unique desires and motivations of academic physicians in Nigeria is important considering the high level of burnout. Exploring these characteristics must be done in a holistic manner. While quantitative surveys can quatify the magnitude of specific motivators and desires, qualitative methods provide nuanced insights into the underline reasons and personal experiences. Such approaches not only help in unveiling the challenges faced in balancing clinical, teaching and research responsibilities, they can also help us to understand the cultural, religious, and socio-economic factors that shape these motivations and desires in the Nigerian context. Ultimately, unveiling these factors may help inform interventions tailored to the specific needs of the Nigerian academic physician.

## Recommendations

The high prevalence of burnout among academic physicians in Nigeria highlights the need for further research and implementation of preventive measures. Based on the findings of this study, some recommendations include:

1. Further examination of the impact of religion on burnout in the Nigerian context, particularly the effect of religious affiliation and self-perception as an active participant in religion on burnout.
2. Further investigation into the factors contributing to the higher incidence of emotional exhaustion among physicians working in Federal government-owned hospitals and those practicing in the North.
3. Encourage participation in enjoyable activities and meaningful work to provide a protective effect against burnout.
4. Further exploration of the unexpected findings, such as the positive effect of age and the negative effect of teaching hours per week on burnout, to better understand the dynamics of burnout among academic physicians in Nigeria.
5. The development and implementation of targeted interventions to increase job satisfaction, such as salary increase and the provision of improved patient care facilities.

## Limitations

This study has certain limitations that should be considered. Firstly, being an online-based, self-administered survey has its inherent limitations, as previously reported [35, 36]. However, these limitations are mitigated by the use of WhatsApp’s broadcast feature to distribute the survey, which allowed us to calculate the response rate, and by the fact that the study population is a professional group, which reduces, to some extent, response bias [35]. Secondly, the timing of the survey coincided with a strike, which could have impacted both the prevalence of burnout and characteristics in federal government and non-federal government-owned hospitals. Thirdly, we used the Maslach Burnout Inventory for Education to measure burnout specifically in academic physicians, some respondents may have found it challenging to distinguish between their roles as physicians and educators, which could introduce some level of bias. However, the reliability score suggests that this may not have been a major factor. Finally, obtaining information about non-responders is a recognized limitation of online and WhatsApp-based surveys, increasing the likelihood of selection bias [36].

## Data Availability

The datasets analysed during the current study are available in the Open Science Framework (OSF) repository Website [14].

## References

[1] Maslach C, Jackson S, Leiter M. (1997) The Maslach Burnout Inventory Manual. In: Evaluating Stress: A Book of Resources. pp 191–218.

[2] Golub JS, Johns MM, Weiss PS, Ramesh AK, Ossoff RH (2008). Burnout in Academic Faculty of Otolaryngology-Head and Neck Surgery. Laryngoscope.

[3] Byrne BM (1991). The Maslach Burnout Inventory: validating Factorial structure and Invariance Across Intermediate, secondary, and University educators. Multivar Behav Res.

[4] Nwosu C, Adeyemi T, Salawu O, Mejabi J, Fadimu A (2018). Orthopaedic implant removal: epidemiology and outcome analysis. Niger J Orthop Trauma.

[5] Chesak SS, Cutshall S, Anderson A, Pulos B, Moeschler S, Bhagra A (2020). Burnout among women Physicians: a call to action. Curr Cardiol Rep.

[6] Ogunsuji OO, Adebayo O, Olaopa O (2019). Burnout among Nigerian doctors: a systematic review. Nigerian Med Practitioner.

[7] Dubale BW, Friedman LE, Chemali Z, Denninger JW, Mehta DH, Alem A, Fricchione GL, Dossett ML, Gelaye B (2019). Systematic review of burnout among healthcare providers in sub-saharan Africa. BMC Public Health.

[8] Elbarazi I, Loney T, Yousef S, Elias A (2017). Prevalence of and factors associated with burnout among health care professionals in arab countries: a systematic review. BMC Health Serv Res.

[9] Lee RT, Seo B, Hladkyj S, Lovell BL, Schwartzmann L (2013). Correlates of physician burnout across regions and specialties: a meta-analysis. Hum Resour Health.

[10] Schmidt GB, Park G, Keeney J, Ghumman S (2017). Job apathy: scale development and initial validation. J Career Assess.

[11] Burn-Out. - Stages of Disillusionment in the Helping Professions | Office of Justice Programs. https://www.ojp.gov/ncjrs/virtual-library/abstracts/burn-out-stages-disillusionment-helping-professions. Accessed 8 Jan 2023.

[12] Harris PL (1984). Assessing burnout: the organizational and individual perspective. Fam Community Health.

[13] Nwosu ADG, Ossai EN, Mba UC, Anikwe I, Ewah R, Obande BO, Achor JU (2020). Physician burnout in Nigeria: a multicentre, cross-sectional study. BMC Health Serv Res.

[14] Ozumba L, Alabere I (2019). Burnout among doctors and nurses at university of Port Harcourt Teaching Hospital, South-South Nigeria. Arch Med Health Sci.

[15] Adebayo O, Olaopa O, Sokomba A, Ogunsuji O, Efuntoye O, Oiwoh S, Atitlola O, Adeoye MA (2019). The burden of burnout and effect on Cardiovascular Diseases and risk predisposition: a call for action. Niger J Med.

[16] Győrffy Z, Dweik D, Girasek E (2018). Willingness to migrate—a potential effect of burnout? A survey of Hungarian physicians. Hum Resour Health.

[17] Ogundipe OA, Olagunju AT, Lasebikan VO, Coker AO (2014). Burnout among doctors in residency training in a tertiary hospital. Asian J Psychiatry.

[18] Coker A, Omoluabi P (2009). Validation of Maslach Burnout Inventory. Ife Psychologia.

[19] Oluwadiya KS, Olasinde AA, Omowumi OI. (2023) Burnout among academic physicians in Nigeria. 10.1186/s12913-023-10366-1 PMC10699013 38053092

[20] Ogboghodo E, Edema O (2020). Assessment of burnout amongst resident doctors in Benin City, Edo State, Nigeria. Niger Postgrad Med J.

[21] Coker AO, Adewole OA, Shoga MO, Uzodimma CC (2012). Burnout syndrome among orthopaedic surgeons in Lagos, Nigeria. East and Central African. J Surg.

[22] Pindar SK, Coker AO, Wakil MA, Morakinyo O, Coker AO (2012). Comparison of burnout syndrome among clinical and non-clinical staff of two tertiary health institutions in Maiduguri, Nigeria. Transnatl J Sci Technol.

[23] Ayinde OO, Akinnuoye ER, Molodynski A, Battrick O, Gureje O (2022). A descriptive study of mental health and burnout among Nigerian medical students. Int J Soc Psychiatry.

[24] Al-Dubai SAR, Rampal KG (2010). Prevalence and Associated factors of burnout among doctors in Yemen. J Occup Health.

[25] Watts J, Robertson N (2011). Burnout in university teaching staff: a systematic literature review. Educational Res.

[26] De Diego-Cordero R, Iglesias-Romo M, Badanta B, Lucchetti G, Vega-Escaño J (2022). Burnout and spirituality among nurses: a scoping review. EXPLORE.

[27] Kaur D, Sambasivan M, Kumar N (2013). Effect of spiritual intelligence, emotional intelligence, psychological ownership and burnout on caring behaviour of nurses: a cross-sectional study. J Clin Nurs.

[28] Haghnegahdar M, Sharma P, Hubbard KP, White WA (2021). The influence of Religious Belief on Burnout in Medical Students. Mo Med.

[29] Lar-Ndam N, Madaki JKA, Pitmang L, Audu MD, Salihu D, Gyang M (2015). Burnout among primary care physicians in Jos-Plateau, north-central Nigeria. Nigerian J Family Pract.

[30] Norvell JG, Baker AM, Carlberg DJ (2021). Does academic practice protect emergency physicians against burnout?. J Am Coll Emerg Physicians Open.

[31] Zhuang C, Hu X, Dill MJ (2022). Do physicians with academic affiliation have lower burnout and higher career-related satisfaction?. BMC Med Educ.

[32] Tijdink JK, Vergouwen ACM, Smulders YM (2013). Publication pressure and burn out among Dutch Medical professors: a Nationwide Survey. PLoS ONE.

[33] Shanafelt TD, West CP, Sloan JA, Novotny PJ, Poland GA, Menaker R, Rummans TA, Dyrbye LN (2009). Career Fit and Burnout among Academic Faculty. Arch Intern Med.

[34] Fernet C, Guay F, Senécal C (2004). Adjusting to job demands: the role of work self-determination and job control in predicting burnout. J Vocat Behav.

[35] Andrade C (2020). The limitations of online surveys. Indian J Psychol Med.

[36] Ameen S, Praharaj SK (2020). Problems in using WhatsApp groups for survey research. Indian J Psychiatry.

